# Different tumor growth pattern of clinically nonfunctioning pituitary neuroendocrine tumor according to sex and age: a longitudinal study

**DOI:** 10.1007/s40618-024-02303-8

**Published:** 2024-02-04

**Authors:** S. S. Park, H. Kang, Y. H. Kim, J. H. Kim

**Affiliations:** 1 Department of Internal Medicine, Seoul National University College of Medicine, 101 Dae-hak ro, Seoul, 03080 Korea; 2 Department of Internal Medicine, Seoul National University Hospital, Seoul, Korea; 3 Pituitary Center, Seoul National University Hospital, Seoul, Korea; 4 Department of Neurosurgery, Seoul National University Hospital, Seoul National University College of Medicine, 101 Dae-hak ro, Seoul, 03080 Korea

**Keywords:** Pituitary neuroendocrine tumor, Non-functioning pituitary neuroendocrine tumor, Age, Sex

## Abstract

**Purpose:**

Asymptomatic patients with clinically non-functional pituitary neuroendocrine tumors (CNF-PitNETs) are usually followed up. However, the natural course of CNF-PitNETs according to sex and age remains unclear. Therefore, this study assessed growth patterns of CNF-PitNETs according to sex and age.

**Methods:**

In this longitudinal study, we enrolled 431 consecutive patients with CNF-PitNETs who were treated at Seoul National University Hospital from 1997 to 2021. The patients underwent hormone function testing and visual field testing, and were subsequently followed up with imaging over a median duration of 66 months.

**Results:**

The median age of the patients was 53.0 years, and 37.1% (*n* = 160) were men. Men were older and harbored more macroadenomas than women. The annual tumor volume change was higher in men than in women (0.21 vs. 0.04 cm^3^/year, *P* < 0.001). The estimated cutoff value of age for significant tumor growth was 51 years. In men, the annual tumor volume change was similar across all age groups. In women, those aged ≤ 50 years showed significantly lower annual tumor volume change than those aged > 50 years (0.01, 0.11, and 0.17 cm^3^/year, *P* = 0.001). When comparing sexes within the same age group, the annual tumor volume changes was significantly lower for women than for men, only in patients aged ≤ 50 years (0.01 vs. 0.15 cm^3^/year, *P* < 0.001).

**Conclusions:**

Among patients with CNF-PitNET, tumor growth was slower in women aged ≤ 50 years than in men and women aged > 50. These findings may guide the customization of surveillance strategies for CNF-PitNETs according to sex and age.

**Supplementary Information:**

The online version contains supplementary material available at 10.1007/s40618-024-02303-8.

## Introduction

Clinically non-functioning pituitary neuroendocrine tumors (CNF-PitNETs) are benign tumors that originate from adenohypophyseal cells. They are characterized by the absence of clinical and biochemical evidence of hormone hypersecretion [1]. CNF-PitNETs account for approximately 15–50% of all pituitary neuroendocrine tumors (PitNETs) [2]; however, the predominant subtype of PA varies depending on sex and age [3]. For instance, prolactinoma predominantly affects women, but CNF-PitNET shows no sex-related bias. The incidence of CNF-PitNET peaks between 50 and 70 years of age, whereas prolactinoma is the most common in the 20–30-year age group [4–6].

Patients with functioning pituitary neuroendocrine tumors typically require surgery or medical treatment at the time of diagnosis. However, close observation may be another option for patients with CNF-PitNET who do not exhibit any signs of mass effects, such as visual field defects [7–9]. Therefore, closely monitoring for any new symptoms that may arise from CNF-PitNET is imperative. Some studies suggest that initial tumor size and the proximity of CNF-PitNET to the optic chiasm serve as prognostic indicators of rapid tumor growth [9–11]. Sam et al. conducted a retrospective study on 66 patients with CNF-PitNET and reported that 73% of macroadenomas in contact with the optic chiasm showed tumor growth, which was significantly higher than the 29% observed for macroadenomas not in contact with the optic chiasm [11]. Karavitaki et al. further confirmed that the annual growth rate of macroadenomas is significantly higher than that of microadenomas [10]. In line with this, Hordejuk et al. [12] and Han et al. [13] suggested that less frequent surveillance may be sufficient for pituitary microadenomas, given slow growth rate of these tumors and the relatively benign natural course. However, the limited knowledge on clinical characteristics associated with rapid tumor growth poses a challenge in customizing and individualizing the follow-up protocol for patients with CNF-PitNET.

While the incidence of CNF-PitNET is similar for men and women, men are generally diagnosed at an older age and with larger tumors [14–16]. Additionally, although age is an independent factor associated with the growth of various tumors [17–19], only few studies have examined on whether CNF-PitNET growth rates vary according to age. Furthermore, data on the natural course of CNF-PitNET in relation sex and age are limited. Therefore, this study aimed to assess whether CNF-PitNET growth patterns differ according to sex and age.

## Materials and methods

### Study subjects

In this longitudinal observational study, we enrolled a total of 431 consecutive patients diagnosed with CNF-PitNET from 1997 to 2021 at Seoul National University Hospital (SNUH). Patients were included if the met the following criteria: (1) imaging features compatible with pituitary adenomas (PAs), (2) lack of clinical and biochemical evidence of hormone hypersecretion, (3) at least 1 year of follow-up without any treatment at the time of diagnosis, and (4) at least two magnetic resonance imaging (MRI) studies during the follow-up period. Patients were excluded if they had (1) imaging features favoring Rathke's cleft cyst or craniopharyngioma, (2) a previous history of CNF-PitNET treatment such as surgery or radiation therapy, or (3) baseline visual field defect or pituitary apoplexy caused by the mass effect of CNF-PitNET. The optimal age cutoff for significant tumor growth was determined using the maximal chi-square method. The cutoff age at diagnosis was estimated to be 51 years. Individuals aged ≥ 65 years were categorized as “elderly”. Consequently, patients were divided into three groups based on their age at diagnosis: Group I (≤ 50 years), Group II (between 51 and 64 years), and Group III (≥ 65 years). Radiological and hormonal characteristics were compared across different age groups and genders.

The follow-up duration was defined as the period from the date of MRI at the time of initial diagnosis to the date of the last MRI evaluation. For patients who underwent transsphenoidal surgery, the date of the most recent MRI scan before surgery was defined as the last follow-up date. This study adhered to the STROBE guidelines and was conducted in accordance with the Helsinki Declaration, receiving approval from the Institutional Review Boards of SNUH (No. 1503-040-654). Written informed consent was obtained from 252 patients who were enrolled prospectively, whereas its requirement was waived for 179 patients who were enrolled retrospectively.

### Hormone assessment

All hormone assessments were performed between 8:00 and 10:00 AM using radioimmunoassay or immunoradiometric assay. Adrenocorticotropic hormone (ACTH), serum cortisol, thyroid-stimulating hormone (TSH), free T4, luteinizing hormone (LH), follicle-stimulating hormone (FSH), estradiol, and total testosterone were measured at the time of diagnosis, and annually during follow-up. Endocrine function was evaluated at the time of diagnosis and on the date of the last follow-up.

ACTH deficiency was defined as a peak cortisol level ≤ 18 μg/dL following the short Synacthen test (SST). In the absence of the SST, ACTH deficiency was defined as morning cortisol levels < 5 μg/dL and low to normal ACTH levels (reference range 10–65 pg/mL). TSH deficiency was defined as a low free T4 level (< 0.70 ng/dL) and low to normal TSH level (reference range 0.4–4.1 mIU/mL). Gonadotropin deficiency was defined in premenopausal women as presenting with oligomenorrhea or amenorrhea, estradiol levels < 50 pg/mL, and low to normal FSH/LH levels. In postmenopausal women, it was defined by estradiol levels < 50 pg/mL and low to normal FSH/LH. Gonadotropin deficiency in men was defined as low testosterone levels with low-to-normal FSH/LH.

An increased number of pituitary hormone deficiencies during follow-up was defined as aggravated endocrine dysfunction, while a decreased number was defined as improved endocrine dysfunction.

### Ophthalmological assessment

Visual field and visual acuity examinations were conducted at the initial diagnosis, six months later, and annually after that. The Humphrey visual field analyzer or Goldmann visual field tests were used to assess the visual field. Subjective visual symptoms were documented for patients who did not undergo visual acuity and visual field examinations during their visits to the outpatient clinic.

### Radiological assessment

MRI was performed at the time of initial diagnosis and then annually for two years. Patients who experienced changes in tumor size during the first two years underwent MRI at 1-year intervals. Conversely, patients without changes in tumor size underwent MRI at the 2-year intervals. MRI and ophthalmological assessments were performed promptly whenever a patient reported with visual disturbances during follow-up. In case of visual disturbance caused by CNF-PitNETs, surgical intervention was considered.

Two investigators independently reviewed all MRI scans and measured the following parameters: (1) cavernous sinus invasion and Knosp Grade (coronal T1-weighted MRI), (2) the shortest distance between the top of the mass and the optic chiasm (coronal T2-weighted MRI), (3) solid or cystic tumor (cystic portion > 50% was defined as a cystic tumor, coronal T2-weighted MRI), (4) macro- and microadenoma (maximal diameter > 1 cm was categorized as macroadenoma, coronal, sagittal, and axial T2-weighted MRI), and (5) tumor volume (coronal T2-weighted coronal MRI) [5].

Tumor volume was calculated by multiplying the tumor area measurement of each coronal view and the slice thickness from the series of segmented tumor slices from an MRI scan. Significant tumor growth was defined as a last follow-up tumor volume was > 120% of the initial tumor volume, following established definitions in previous studies [5, 20]. When the last follow-up tumor volume less than 80% of the initial tumor volume, it was categorized as a decreased tumor. A stable tumor was defined as a volume change between 80 and 120% of the initial tumor volume.

### Statistical analysis

All variables are presented as medians (interquartile ranges) for continuous variables and numbers (%) for categorical variables. The Mann–Whitney *U* test was used to compare continuous variables between two groups, whereas Chi-square test was used for the categorical variables. Maxstat, a maximal chi-square method, was used to identify the optimal cutoff value for age in predicting significant tumor growth. Differences in characteristics between the three age groups were compared using the Kuskal-Wallis test. A *P*-value less than 0.05 was considered statistically significant. The hazard ratios (HRs) and 95% confidence intervals (CIs) for significant tumor growth and the composite outcome were calculated for each variable using the Cox-proportional hazard model. All statistical analyses were performed using R statistical software (Foundation for Statistical Computing, Vienna, Austria, version 4.1.2) and SPSS (IBM, Armonk, NY, USA, version 26).

## Results

Table 1 presents the baseline characteristics of the 431 patients enrolled in this study. The proportion of women in the cohort was 62.9% (n = 271), which was higher than that of men. The median age of men was 58.0 years, which was significantly older than the median age of 50.0 years in women (*P* < 0.001). Men were more likely to have macroadenomas and tumors located less than 1 mm from the optic chiasm compared to women, with 91.9% of men having macroadenomas compared with the 71.2% in women, and 56.2% of men's tumors being less than 1 mm from the chiasm compared with the 36.5% of women’s tumors. All these differences were statistically significant (P < 0.001). Tumor volume at diagnosis was significantly larger in men than in women (2.05 vs. 1.30 cm^3^, *P* < 0.001). Men exhibited fewer cystic tumors than women (18.8% vs. 28.4%, *P* = 0.033). The proportion of tumors with cavernous sinus invasion did not differ significantly between sexes, but the Knosp grade distribution differed significantly. Regarding the hormone status at diagnosis, TSH deficiency was more prevalent in men than in women (8.3% vs. 1.6%, *P* = 0.002). However, there was no significant difference in the total number of hormone deficits observed between the sexes.

**Table 1:** Baseline characteristics of subjects with nonfunctioning pituitary adenomas according to sex

	Male (*n* = 160)	Female (*n* = 271)	Total (*n* = 431)	*P* value
Age (years)	58.0 [47.0;65.0]	50.0 [39.0;60.0]	53.0 [42.0;62.0]	< 0.001
Macroadenoma	147 (91.9%)	193 (71.2%)	340 (78.9%)	< 0.001
Tumor volume at diagnosis (cm^3^)	2.05 [1.16;4.13]	1.30 [0.61;2.58]	1.59 [0.76;2.93]	< 0.001
Cystic tumor	30 (18.8%)	77 (28.4%)	107 (24.8%)	0.033
Tumors distant from optic chiasm < 1 mm	90 (56.2%)	99 (36.5%)	189 (43.9%)	< 0.001
Cavernous sinus invasion	25 (15.6%)	40 (14.8%)	65 (15.1%)	0.918
Knosp grade				0.012
Grade 0	46 (28.8%)	112 (41.3%)	158 (36.7%)	
Grade 1	50 (31.2%)	76 (28.0%)	126 (29.2%)	
Grade 2	39 (24.4%)	43 (15.9%)	82 (19.0%)	
Grade 3	22 (13.8%)	26 (9.6%)	48 (11.1%)	
Grade 4	3 (1.9%)	14 (5.2%)	17 (3.9%)	
Hormone deficiency at diagnosis				
TSH deficiency	13 (8.3%)	4 (1.6%)	17 (4.1%)	0.002
ACTH deficiency	19 (12.1%)	32 (12.7%)	51 (12.5%)	0.969
Gonadotropin deficiency	27 (19.6%)	45 (17.0%)	72 (17.9%)	0.625
Total number of hormone deficit				0.187
0	114 (71.2%)	198 (73.1%)	312 (72.4%)	
1	35 (21.9%)	66 (24.4%)	101 (23.4%)	
2	9 (5.6%)	6 (2.2%)	15 (3.5%)	
3	2 (1.2%)	1 (0.4%)	3 (0.7%)	
Annual tumor volume change (cm^3^/year)	0.21 [0.02;0.70]	0.04 [– 0.02;0.28]	0.09 [– 0.01;0.42]	< 0.001
Annual tumor volume percent change (%/year)	10.9 [ 1.2;27.9]	5.2 [– 2.4;20.7]	6.7 [– 1.3;23.3]	0.001
Tumor volume growth ≥ 20%	105 (65.6%)	141 (52.0%)	246 (57.1%)	0.008
Worsening of visual function	18 (18.2%)	12 (11.0%)	30 (14.4%)	0.203
Worsening of endocrine dysfunction	12 (7.5%)	19 (7.0%)	31 (7.2%)	1.000
Surgery during follow-up	42 (26.2%)	31 (11.5%)	73 (17.0%)	< 0.001
Follow-up duration (months)	61.5 [37.0;101.5]	71.0 [38.5;97.0]	66.0 [38.0;98.0]	0.677

Data are presented as mean ± standard deviation or *n* (%). Mann–Whitney *U* test was used for continuous variables and Chi-square test was used for categorical variables. Cystic tumor was evaluated by cystic portion of tumor > 50%

*TSH* thyroid stimulating hormone, *ACTH* adrenocorticotropic hormone

**Fig. 1:**
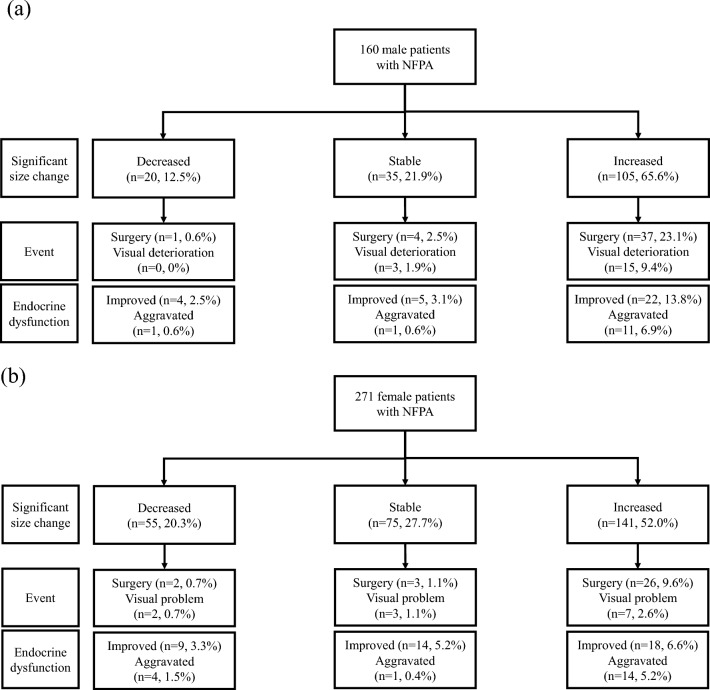
The natural course of **a** males and **b** females with nonfunctioning pituitary adenoma

Figure 1 illustrates the progression of CNF-PitNET in patients over a 66-month follow-up period. In men, tumor size increased in 65.6% and decreased in 12.5%, whereas in women, these proportions were 52.5% and 20.3%, respectively. Visual symptoms were more common in men (11.3%) than in women (4.4%). Endocrine dysfunction worsened similarly in both sexes (13.8% in men and 14.1% in women). Among men experiencing tumor growth (*n* = 105), 37 underwent surgery, primarily due to visual deterioration (*n* = 15). In women with significant tumor growth (*n* = 141), 26 underwent surgery, with 7 citing visual deterioration.

Table 1 shows that men exhibited higher annual tumor volume change and percent change than women (0.21 vs. 0.04 cm^3^/year and 10.9% vs. 5.2%, respectively, all *P* < 0.01). During similar follow-up duration in both sexes (61.5 vs. 71.0 months,* P* = 0.677), men experienced more significant tumor growth (volume increase ≥ 20%) than women (65.6% vs. 52.0%, *P* = 0.002). More men underwent surgery than women (26.2% vs. 11.5%, *P* < 0.001), while the worsening of visual function and endocrine dysfunction occurred similarly in both sexes.

**Fig. 2:**
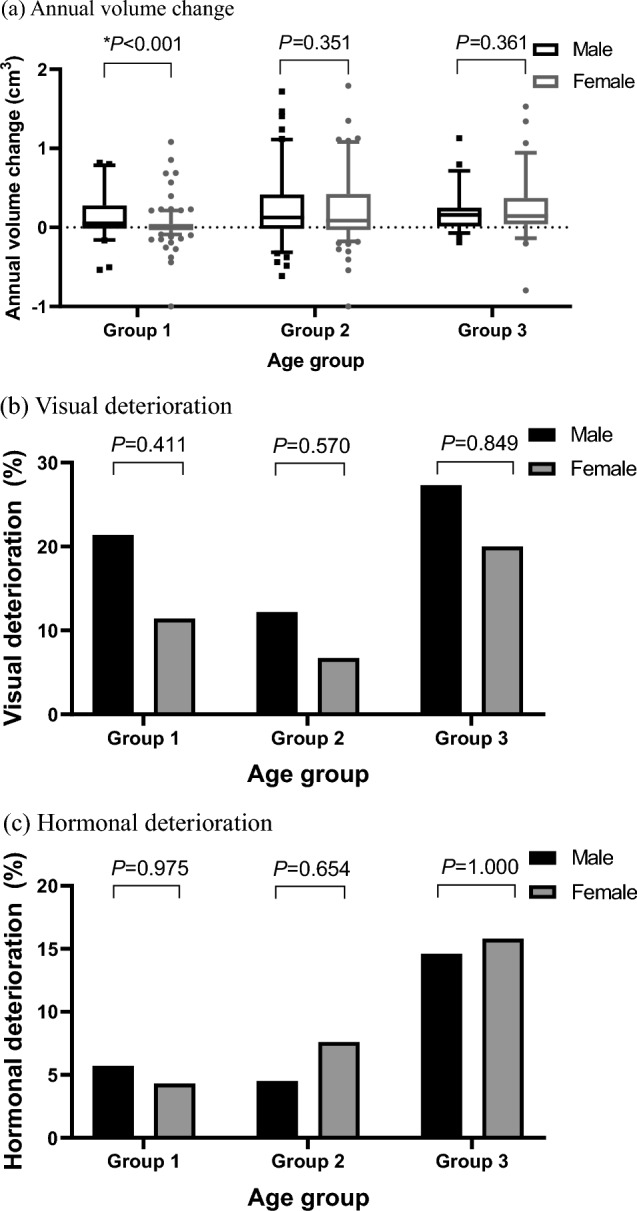
Sex-specific **a** tumor volume change, **b** visual deterioration, **c** hormonal deterioration in patients with nonfunctioning pituitary adenoma according to age groups

Table 2 shows the baseline characteristics and the natural course of CNF-PitNET patients according to age groups in (a) men and (b) women. The highest incidence of macroadenomas and the largest tumor volumes at diagnosis were found in the oldest age group (Group III) for both sexes. The youngest age group (Group I) showed smaller tumor sizes at diagnosis and fewer cases of tumors invading the cavernous sinus compared with that in the other age groups in both men and women. In women, the annual increase in tumor volume (0.01 vs. 0.11 vs. 0.17 cm^3^/year, *P* = 0.001) and the percentage of tumor growth (2.3% vs. 6.7% vs. 10.1%/year, *P* = 0.018) were significantly greater in the oldest age group (Group III). However, these changes were not significantly different across age groups in men. A similar trend was identified in patients with pituitary macroadenomas, as shown in Table 3. Figure 2a demonstrates that tumor growth rates were higher in men than in women across all age groups. Notably, in the youngest group of women (Group I), tumor volume remained relatively stable over 76 months. Meanwhile, significant tumor growth was more common in the oldest group of women (Group III) (44.0% vs. 58.7% vs. 65.8%, *P* = 0.027), even though this group had a shorter follow-up period (76.0 vs. 65.5 vs. 55.5 months, *P* = 0.014). In both men and women, no significant differences were noted in the worsening of visual dysfunction across all age groups. In men, no significant differences were found in the worsening of endocrine dysfunction across all age groups. However, in women, a significant difference was noted in the worsening of endocrine dysfunction across age groups, with more significant deterioration observed in older age groups. In each age group, there was no significant difference between men and women in terms of visual or hormonal deterioration (Fig. 2b, c). We found no significant differences in the worsening of visual functions or endocrine dysfunction across age groups in either sex in patients with pituitary macroadenomas, as shown in Table 3.

**Table 2:** Baseline characteristics and natural course of (a) males and (b) females with nonfunctioning pituitary adenoma according to age

(a)				
Variables	male ≤ 50 years (*n* = 53)	50 < male < 65 years (*n* = 66)	male ≥ 65 years (*n* = 41)	*P* value
Age at diagnosis	43.0 [33.0;47.0]	59.0 [55.0;62.0]	68.0 [67.0;72.0]	< 0.001
Macroadenoma	44 (83.0%)	62 (93.9%)	41 (100.0%)	0.008
Tumor volume at diagnosis (cm^3^)	1.81 [0.72;2.92]	2.22 [1.41;4.53]	2.47 [1.38;4.85]	0.018
Cystic tumor	14 (26.4%)	12 (18.2%)	4 (9.8%)	0.120
Tumors distant from optic chiasm < 1mm	27 (50.9%)	40 (60.6%)	23 (56.1%)	0.572
Cavernous sinus invasion	1 (1.9%)	16 (24.2%)	8 (19.5%)	0.003
Knosp grade				< 0.001
Grade 0	26 (49.1%)	12 (18.2%)	8 (19.5%)	
Grade 1	14 (26.4%)	24 (36.4%)	12 (29.3%)	
Grade 2	12 (22.6%)	14 (21.2%)	13 (31.7%)	
Grade 3	1 (1.9%)	16 (24.2%)	5 (12.2%)	
Grade 4	0 (0.0%)	0 (0.0%)	3 (7.3%)	
Hormone deficiency at diagnosis				
TSH deficiency	3 (5.8%)	5 (7.7%)	5 (12.5%)	0.497
ACTH deficiency	6 (11.3%)	8 (12.7%)	5 (12.2%)	0.974
Gonadotropin deficiency	11 (22.0%)	8 (15.4%)	8 (22.2%)	0.629
Total number of hormone deficit				0.841
0	36 (67.9%)	50 (75.8%)	28 (68.3%)	
1	14 (26.4%)	12 (18.2%)	9 (22.0%)	
2	3 (5.7%)	3 (4.5%)	3 (7.3%)	
3	0 (0.0%)	1 (1.5%)	1 (2.4%)	
Annual tumor volume change (cm^3^/year)	0.15 [0.01;0.53]	0.19 [0.02;0.93]	0.24 [0.07;0.71]	0.702
Annual tumor volume percent change (%/year)	9.2 [1.3;32.5]	11.9 [1.2;27.4]	11.2 [1.0;22.0]	0.979
Tumor volume growth ≥ 20%	34 (64.2%)	45 (68.2%)	26 (63.4%)	0.847
Worsening of visual function	6 (21.4%)	6 (12.2%)	6 (27.3%)	0.275
Worsening of endocrine dysfunction	3 (5.7%)	3 (4.5%)	6 (14.6%)	0.129
Surgery during follow-up	19 (35.8%)	11 (16.7%)	12 (29.3%)	0.054
Follow-up duration (months)	66.0 [42.0;107.0]	61.5 [37.0;96.0]	58.0 [37.0;85.0]	0.528

(b)				
Variables	female ≤ 50 years (*n* = 141)	50 < female < 65 years (*n* = 92)	female ≥ 65 years (*n* = 38)	*P* value
Age at diagnosis	39.0 [33.0;46.0]	58.0 [55.0;61.0]	70.0 [67.0;72.0]	< 0.001
Macroadenoma	83 (58.9%)	75 (81.5%)	35 (92.1%)	< 0.001
Tumor volume at diagnosis (cm^3^)	0.79 [0.43;1.61]	2.18 [1.04;3.31]	2.15 [1.35;2.82]	< 0.001
Cystic tumor	46 (32.6%)	23 (25.0%)	8 (21.1%)	0.251
Tumors distant from optic chiasm < 1mm	41 (29.1%)	41 (44.6%)	17 (44.7%)	0.030
Cavernous sinus invasion	11 (7.8%)	18 (19.6%)	11 (28.9%)	0.001
Knosp grade				< 0.001
Grade 0	75 (53.2%)	28 (30.4%)	9 (23.7%)	
Grade 1	44 (31.2%)	25 (27.2%)	7 (18.4%)	
Grade 2	11 (7.8%)	21 (22.8%)	11 (28.9%)	
Grade 3	7 (5.0%)	11 (12.0%)	8 (21.1%)	
Grade 4	4 (2.8%)	7 (7.6%)	3 (7.9%)	
Hormone deficiency at diagnosis				
TSH deficiency	3 (2.3%)	1 (1.1%)	0 (0.0%)	0.578
ACTH deficiency	20 (15.5%)	6 (6.9%)	6 (17.1%)	0.125
Gonadotropin deficiency	29 (21.6%)	12 (13.0%)	4 (10.5%)	0.123
Total number of hormone deficit				0.155
0	94 (66.7%)	74 (80.4%)	30 (78.9%)	
1	43 (30.5%)	17 (18.5%)	6 (15.8%)	
2	3 (2.1%)	1 (1.1%)	2 (5.3%)	
3	1 (0.7%)	0 (0.0%)	0 (0.0%)	
Annual tumor volume change (cm^3^/year)	0.01 [– 0.02;0.09]	0.11 [– 0.03;0.51]	0.17 [0.06;0.43]	0.001
Annual tumor volume percent change (%/year)	2.3 [– 3.3;15.3]	6.7 [– 2.1;23.7]	10.1 [ 2.9;25.7]	0.027
Tumor volume growth ≥ 20%	62 (44.0%)	54 (58.7%)	25 (65.8%)	0.017
Worsening of visual function	5 (11.4%)	3 (6.7%)	4 (20.0%)	0.283
Worsening of endocrine dysfunction	6 (4.3%)	7 (7.6%)	6 (15.8%)	0.045
Surgery during follow-up	17 (12.1%)	9 (9.8%)	5 (13.2%)	0.808
Follow-up duration (months)	76.0 [47.0;99.0]	65.5 [38.0;88.0]	55.5 [17.0;92.0]	0.014

Data are presented as mean ± standard deviation or *n* (%). Kruskal wallis test was used for continuous variables and Chi-square test was used for categorical variables. Cystic tumor was evaluated by cystic portion of tumor > 50%

*TSH* thyroid stimulating hormone, *ACTH* adrenocorticotropic hormone

Data are presented as mean ± standard deviation or n (%). Kruskal wallis test was used for continuous variables and Chi-square test was used for categorical variables. Cystic tumor was evaluated by cystic portion of tumor > 50%. TSH, thyroid stimulating hormone; ACTH, adrenocorticotropic hormone

**Table 3:** Baseline characteristics and natural course of (a) males and (b) females with nonfunctioning pituitary macroadenoma according to age

(a)				
Variables	male ≤ 50 years (*n* = 44)	50 < male < 65 years (*n* = 62)	male ≥ 65 years (*n* = 41)	*P* value
Age at diagnosis	43.5 [33.0;46.5]	59.5 [55.0;62.0]	68.0 [67.0;72.0]	< 0.001
Tumor volume at diagnosis (cm^3^)	2.14 [1.12;3.29]	2.39 [1.53;4.54]	2.47 [1.38;4.85]	0.279
Cystic tumor	12 (27.3%)	11 (17.7%)	4 (9.8%)	0.112
Tumors distant from optic chiasm < 1mm	26 (59.1%)	40 (64.5%)	23 (56.1%)	0.674
Cavernous sinus invasion	1 (2.3%)	16 (25.8%)	8 (19.5%)	0.006
Knosp grade				< 0.001
Grade 0	19 (43.2%)	9 (14.5%)	8 (19.5%)	
Grade 1	13 (29.5%)	23 (37.1%)	12 (29.3%)	
Grade 2	11 (25.0%)	14 (22.6%)	13 (31.7%)	
Grade 3	1 (2.3%)	16 (25.8%)	5 (12.2%)	
Grade 4	0 (0.0%)	0 (0.0%)	3 (7.3%)	
Hormone deficiency at diagnosis				
TSH deficiency	2 (4.7%)	5 (8.2%)	5 (12.5%)	0.433
ACTH deficiency	6 (13.6%)	8 (13.6%)	5 (12.2%)	0.975
Gonadotropin deficiency	10 (23.3%)	8 (15.7%)	8 (22.2%)	0.610
Annual tumor volume change (cm^3^/year)	0.26 [0.04;0.73]	0.22 [0.02;0.93]	0.24 [0.07;0.71]	0.947
Annual tumor volume percent change (%/year)	10.3 [ 2.9;32.9]	14.4 [ 1.1;27.4]	11.2 [ 1.0;22.0]	0.957
Tumor volume growth ≥ 20%	30 (68.2%)	43 (69.4%)	26 (63.4%)	0.812
Worsening of visual function	4 (15.4%)	6 (12.8%)	6 (27.3%)	0.316
Worsening of endocrine dysfunction	2 (4.5%)	2 (3.2%)	6 (14.6%)	0.062
Surgery during follow-up	17 (38.6%)	10 (16.1%)	12 (29.3%)	0.032
Follow-up duration (months)	61.5 [37.5;102.0]	61.5 [37.0;96.0]	58.0 [37.0;85.0]	0.842

(b)				
Variables	female ≤ 50 years (*n* = 83)	50 < female < 65 years (*n* = 75)	female ≥ 65 years (*n* = 35)	*P* value
Age at diagnosis	42.0 [34.5;47.0]	58.0 [56.0;61.0]	70.0 [67.0;72.0]	< 0.001
Tumor volume at diagnosis (cm^3^)	1.33 [0.86;2.37]	2.51 [1.66;3.87]	2.24 [1.59;3.16]	< 0.001
Cystic tumor	27 (32.5%)	19 (25.3%)	7 (20.0%)	0.330
Tumors distant from optic chiasm < 1mm	40 (48.2%)	41 (54.7%)	17 (48.6%)	0.689
Cavernous sinus invasion	11 (13.3%)	17 (22.7%)	11 (31.4%)	0.064
Knosp grade				0.012
Grade 0	33 (39.8%)	16 (21.3%)	6 (17.1%)	
Grade 1	29 (34.9%)	22 (29.3%)	7 (20.0%)	
Grade 2	10 (12.0%)	20 (26.7%)	11 (31.4%)	
Grade 3	7 (8.4%)	10 (13.3%)	8 (22.9%)	
Grade 4	4 (4.8%)	7 (9.3%)	3 (8.6%)	
Hormone deficiency at diagnosis				
TSH deficiency	1 (1.3%)	1 (1.4%)	0 (0.0%)	0.801
ACTH deficiency	13 (16.2%)	3 (4.1%)	5 (15.6%)	0.043
Gonadotropin deficiency	16 (19.5%)	12 (16.0%)	4 (11.4%)	0.551
Annual tumor volume change (cm^3^/year)	0.03 [– 0.04;0.23]	0.20 [– 0.01;0.61]	0.17 [0.08;0.48]	0.037
Annual tumor volume percent change (%/year)	2.4 [– 3.6;15.9]	7.9 [– 0.5;23.7]	10.1 [ 3.9;24.5]	0.075
Tumor volume growth ≥ 20%	37 (44.6%)	46 (61.3%)	23 (65.7%)	0.039
Worsening of visual function	5 (14.3%)	3 (7.1%)	3 (16.7%)	0.469
Worsening of endocrine dysfunction	5 (6.0%)	6 (8.0%)	6 (17.1%)	0.143
Surgery during follow-up	16 (19.5%)	8 (10.7%)	5 (14.3%)	0.299
Follow-up duration (months)	72.0 [44.5;95.0]	62.0 [36.5;86.0]	53.0 [18.5;89.0]	0.070

Data are presented as mean ± standard deviation or *n* (%). Kruskal wallis test was used for continuous variables and Chi-square test was used for categorical variables. Cystic tumor was evaluated by cystic portion of tumor > 50%

*TSH* thyroid stimulating hormone, *ACTH* adrenocorticotropic hormone

The impact on significant tumor growth was analyzed using the Cox proportional hazard model (Supplementary Table 1). The results suggested that proximity to the optic chiasm (< 1 mm) is an independent risk factor for significant tumor growth, whereas the presence of a cystic tumor is associated with a lower risk of such growth (all *P* < 0.05). However, the number of hormone deficits, initial tumor volume, and presence of a microadenoma were not identified as significant risk factors. In men, the HR for significant tumor growth was comparable across different age groups. However, in women, the HR increased with the older age groups (HR 1.42, 95% CI 0.97–2.07 for Group II; HR 2.08, 95% CI 1.28–3.36 for Group III) (Supplementary Table 1).

## Discussion

In this longitudinal observational study, we analyzed the differences in the natural course of CNF-PitNET between men and women within different age groups. The findings indicated that men harbored more macroadenomas and exhibited a faster rate of tumor growth than women. The optimal cutoff age for significant tumor growth was 51 years. This study demonstrated that the annual tumor growth rate varied across age groups, but this variation was observed only among women. In particular, women aged ≤ 50 years (group I) exhibited a smaller initial tumor volume and a slower annual tumor growth rate than women aged > 50 years (Group II and III).

This study demonstrated that men had larger tumor volumes and a higher prevalence of macroadenomas at diagnosis than women. This finding aligns with the results of previous studies that have reported a predominance of macroadenomas in men with CNF-PitNET compared with women [14–16]. For instance, an Italian study of 73 patients with CNF-PitNET, found that the prevalence of macroadenoma was 85.7% in men and 47.3% in women [14]. Similarly, a multicenter retrospective study in Spain involving 189 patients with CNF-PitNET demonstrated higher predominance of macroadenomas in men than in women (85.7% vs. 58.6%) [15]. Additionally, a retrospective study conducted in Italy with 371 patients, revealed that macroadenomas were prevalent among 78.8% of men, and only among 32.6% of women [16]. The present study consistently found a higher prevalence of macroadenomas among men than among women. Notably, the incidence of macroadenomas in this study was higher in both sexes than that reported in other studies [14–16].

The reasons for the sex-related differences in the prevalence of macroadenoma remain unclear. However, considering that the prevalence of macroadenomas tends to increase with age, the older age at diagnosis among men than among women may have contributed to the higher prevalence of macroadenoma in men. The lack of symptoms in patients with CNF-PitNET can lead to a delayed diagnosis compared to other types of functioning pituitary neuroendocrine tumors. Women may be diagnosed earlier than men due to early manifestation of symptoms associated with hypopituitarism, such as menstrual irregularities. Moreover, men often exhibit fewer symptoms of hypopituitarism caused by the mass effect of the tumor, contributing to the relatively delayed diagnosis. Similarly, prolactinomas are often diagnosed later in men than in women, resulting in a more aggressive disease course in many cases [21, 22]. In several studies, men with CNF-PitNET tended to be diagnosed at a relatively older age than women [5, 14, 23]. In the present study, the median age at diagnosis for men was approximately 8 years higher than that for women, which may have contributed to the higher prevalence of macroadenomas in men.

Differences in sex hormones could be another explanation for the observed sex-based disparities in macroadenoma prevalence. Di Somma et al. suggested that sex hormones are crucial in determining the diverse tumor behavior between men and women [14]. Gonadotroph adenomas, the predominant histological subtype of CNF-PitNET, are regulated by negative feedback from estrogen [24–26]. Consequently, the cessation of estrogen’s negative feedback after menopause could contribute to an increase in the size of CNF-PitNET. The influence of sex hormones on CNF-PitNET growth may partially account for the differences in growth rates observed across different age groups in both sexes. In the present study, the tumor growth rate was higher in women > 50 years than in women ≤ 50 years. Among women > 50 years, the risk of significant tumor growth increased and became comparable to the risk in men ≤ 50 years. This could be related to sex hormone changes during the menopausal period in women. Estrogen may inhibit the growth of CNF-PitNETs in premenopausal women [24–26]. However, this effect would be negated after menopause. Additionally, the lack of negative feedback by estrogen in menopausal women could contribute to CNF-PitNET development by stimulating gonadotroph cells. Previous research has reported pituitary hyperplasia caused by gonadotropin-releasing hormone hypersecretion [27]. In contrast, men experience gradual changes in testosterone levels with aging, which may lead to relatively gradual changes in tumor growth. Similar trends were observed even in patients with macroadenoma, further supporting the effect of age on tumor growth among women. However, additional studies are warranted to thoroughly investigate the underlying mechanisms for the different effects of age on tumor growth between sexes.

Whether the treatment strategy for CNF-PitNET should be tailored according to sex and age remains unclear. Several studies have demonstrated that the safety and effectiveness of transsphenoidal surgery in older adults are comparable to those in younger patients. However, hormone recovery after transsphenoidal approach (TSA) was relatively decreased in the older age groups [4, 28–31]. Given the safety and effectiveness of TSA surgery demonstrated in previous studies, close monitoring and appropriate management of CNF-PitNET growth may be necessary, even in older adults. In men, no significant difference was noted in the risk of substantial tumor growth according to age. Conversely, in women, the risk of significant tumor growth remained relatively low until menopause. Therefore, less frequent monitoring of CNF-PitNET growth may be necessary for women ≤ 50 years.

This study has several strengths. First, it is the largest to date, encompassing 431 patients, meticulously evaluating tumor growth rate in surgery-naive patients with CNF-PitNET. Second, the median follow-up duration of 66 months, enabled comprehensive monitoring of the natural course of CNF-PitNETs. Thirdly, this study pioneered the reporting of sex- and age-specific natural courses of CNF-PitNETs.

Despite its strengths, this study is not without limitations. First, its observational nature necessitated comparing tumor growth rates based on age at initial diagnosis, rather than prospectively tracking tumor growth rates over time. This limitation makes it challenging to definitively determine whether tumors grow faster with age or later-diagnosed tumors exhibit accelerated growth. However, it is more likely that tumor growth accelerates with age, as a delayed diagnosis often indicates an indolent disease course. Secondly, this study restricted the comparison to tumor volumes at initial diagnosis and last follow-up. Thirdly, data regarding growth hormone (GH) deficiency was limited due to the relatively older ages of patients with CNF-PitNET, hindering a thorough assessment of GH deficiency.

## Conclusions

This study revealed distinct tumor growth patterns between men and women with CNF-PitNETs. Men exhibited a higher prevalence of macroadenomas and a faster annual tumor volume change than women. Interestingly, no significant difference in annual tumor volume among men across different age groups was observed. In contrast, women aged ≤ 50 years demonstrated significantly slower annual tumor volume change compared to women aged > 50 years. These differential growth patterns suggest that a uniform approach to monitoring CNF-PitNET may not be optimal. Tailoring surveillance strategies based on sex and age could lead to more effective clinical management. For instance, younger women might require less frequent monitoring due to their slower tumor growth rate. Further research is warranted to elucidate the underlying mechanisms responsible for these growth pattern disparities in CNF-PitNET, ultimately deepening the understanding of its nature and potentially identifying novel therapeutic targets.

### Supplementary Information

Below is the link to the electronic supplementary material.

Supplementary file1 (DOCX 16 KB)

## Data Availability

The datasets generated during and analyzed during the current study are available from the corresponding author on reasonable request.
