# Supplementary material for: Different tumor growth pattern of clinically nonfunctioning pituitary neuroendocrine tumor according to sex and age: a longitudinal study
Source: J Endocrinol Invest. 2024 Feb 4;47(8):1911–21. doi: 10.1007/s40618-024-02303-8 (PMC11266204; doi:10.1007/s40618-024-02303-8)
Supplement: Supplementary file 1 — Supplementary file1 (DOCX 16 KB) [file 40618_2024_2303_MOESM1_ESM.docx]

Supplementary **Table 1**.

|  | Univariate | | Multivariate | |
| --- | --- | --- | --- | --- |
|  | HR (95% CI) | *P* | HR (95%CI) | *P* |
| Number of hormone deficit | 0.91 (0.73–1.15) | 0.436 |  |  |
| Proximity to optic chiasm (<1mm) | 1.78 (1.39–2.30) | <0.001 | 1.44 (1.08–1.93) | 0.013 |
| Microadenoma | 0.49 (0.34–0.69) | <0.001 | 0.71 (0.48–1.06) | 0.098 |
| Cystic tumor | 0.67 (0.48–0.93) | 0.017 | 0.70 (0.50-0.97) | 0.034 |
| Initial tumor volume | 1.04 (1.02–1.06) | <0.001 | 1.02 (0.99–1.06) | 0.178 |
| Age group |  |  |  |  |
| Females ≤ 50yrs | Reference |  | Reference |  |
| 50yrs <Females ≤ 65yrs | 1.67 (1.16-2.41) | 0.006 | 1.42 (0.97-2.07) | 0.070 |
| Females ≥65yrs | 2.40 (1.50-3.84) | <0.001 | 2.07 (1.28-3.36) | 0.003 |
| Males ≤ 50yrs | 1.57 (1.03-2.39) | 0.037 | 1.40 (0.91-2.14) | 0.124 |
| 50yrs <Males ≤ 65yrs | 1.91 (1.39-2.81) | 0.001 | 1.49 (0.99-2.23) | 0.053 |
| Males ≥65yrs | 1.87 (1.18-2.97) | 0.008 | 1.27 (0.78-2.06) | 0.327 |

The Cox proportional hazard model was employed to evaluate the hazard ratio for significant tumor growth. Yrs, years;
